# Small Cell Lung Cancer Patient with Profound Hyponatremia and Acute Neurological Symptoms: An Effective Treatment with Fludrocortisone

**DOI:** 10.1155/2015/286029

**Published:** 2015-07-09

**Authors:** Jana Jaal, Tõnu Jõgi, Alan Altraja

**Affiliations:** ^1^Hematology and Oncology Clinic, Department of Radiotherapy and Oncological Therapy, Tartu University Hospital, 51014 Tartu, Estonia; ^2^Department of Haematology and Oncology, University of Tartu, 51014 Tartu, Estonia; ^3^Department of Pulmonary Medicine, University of Tartu, 51014 Tartu, Estonia; ^4^Lung Clinic, Tartu University Hospital, 51014 Tartu, Estonia

## Abstract

Hyponatremia is a frequent electrolyte abnormality in patients with small cell lung cancer (SCLC). Being usually asymptomatic, hyponatremia may cause symptoms like nausea, fatigue, disorientation, headache, muscle cramps, or even seizures, particularly if severe and rapid decrease of serum sodium levels occurs. Here we report a case of SCLC patient with severe hyponatremia and acute neurological symptoms that developed 2 days after the first course of second-line chemotherapy, most probably due to the release of antidiuretic hormone (ADH, also known as arginine vasopressin) during lysis of the tumour cells. Initial treatment consisted of continuous administration of hypertonic saline that resulted in improvement of patient's neurological status. However, to obtain a persistent increase in serum sodium level, pharmacological intervention with oral fludrocortisone 0.1 mg twice daily was needed. We can therefore conclude that mineralocorticoids may be used to correct hyponatremia in SCLC patients when appropriate.

## 1. Introduction

Lung cancer is one of the most frequent and deadly types of cancer worldwide that causes more deaths than breast, colon, and prostate cancer combined [1]. Small cell lung cancer (SCLC) is a histologic subtype with a distinct biology and aggressive clinical course that comprises approximately 15–20% of all cases of lung cancer [2]. Although the overall incidence of SCLC is slightly declining, mainly due to the decrease in the percentage of smokers, it still remains a huge challenge in oncology [2]. Despite the decades of extensive research, the outcome of SCLC patients remains very poor, suggesting the need for more efficacious treatments and improved patient care [3].

Hyponatremia, defined as a serum sodium level of <136 mmol/L, is a frequent electrolyte abnormality in SCLC patients. In previous studies, rates of hyponatremia up to 44-45% have been reported with most cases caused by the paraneoplastic syndrome of inadequate antidiuretic hormone (ADH, also known as arginine vasopressin) secretion (SIADH) [4, 5]. Despite being prevalent in patients with SCLC, the influence of hyponatremia on the prognosis is largely underestimated in clinical practice. The emerging evidence, mainly from large scale retrospective studies, shows that hyponatremia is an independent factor of poor prognosis in patients with SCLC [6–8].

In the majority of cases, hyponatremia is asymptomatic in patients with SCLC. However, it may cause symptoms like nausea, fatigue, disorientation, headache, and muscle cramps or even seizures, particularly if a severe and rapid decrease of serum sodium levels occurs [4, 7]. Moreover, in some patients with SCLC, a delirious state might be the first neurological symptom of the paraneoplastic syndrome and the first sign of the underlying malignant lung disease [9].

Here, we report a case of SCLC patient with severe hyponatremia and acute neurological symptoms that developed 2 days after the first course of second-line chemotherapy, most probably due to the release of ADH during lysis of the tumour cells. Persistent increase in serum sodium levels of this patient was achieved only with fludrocortisone therapy.

## 2. Case Presentation

A 57-year-old male patient with a smoking history of more than 40 years and extensive stage SCLC was brought to The Emergency Department at the Tartu University Hospital due to acute neurological symptoms: the patient had suddenly become disoriented and did not recognize his family members and relatives. Severe hyponatremia was diagnosed at his admission, with the sodium level of 104 mmol/L.

The patient had first been admitted to the Tartu University Hospital approximately 7 months earlier with extensive stage SCLC (lymph node and liver metastases). Patient's serum sodium level at diagnosis was normal. Patient's initial treatment had consisted of 6 cycles of chemotherapy with cisplatin and etoposide that had resulted in stable disease. Afterwards, the patient had been followed up for 2 months. Subsequently, a progressive disease had been diagnosed in primary site and liver and second-line chemotherapy with topotecan started. First cycle of topotecan had ended 2 days prior to the patient's admittance to the emergency department with acute neurological symptoms (mainly disorientation) described above.

In parallel with the hyponatremia referred to above, other abnormal blood tests included slight increases in the levels of serum bilirubin (43 *μ*mol/L) and liver enzymes (alanine aminotransferase 145 U/L, aspartate aminotransferase 107 U/L), as well as increased levels of alkaline phosphatase (460 U/L) and lactate dehydrogenase (798 U/L). Serum levels of potassium, glucose, creatinine, urea, and ammonia were normal. Serum osmolality was decreased (218 mOsm/L), which is a frequent finding in patients with hyponatremia. For unknown reasons, urine sodium and osmolality analyses were not ordered by first physicians taking care of the patient. Definitely, these latter analyses would have added valuable information in the management of this case. Patient was euvolemic.

The computed tomography with contrast media showed no brain metastases.

**Figure 1:**
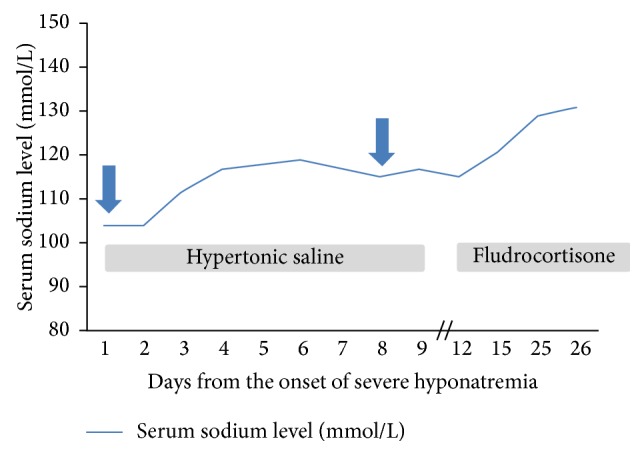
Changes in serum sodium levels during the treatment of hyponatremia in a patient with extensive stage SCLC. The figure illustrates changes in serum sodium levels in relation to the received treatment (hypertonic saline or fludrocortisone). The blue arrows indicate the onset of neurological symptoms. The first and relatively severe neurological symptoms (disorientation, mental confusedness) appeared 2 days after the completion of the first cycle of second-line chemotherapy with topotecan. Secondary, a slighter and less durable disorientation appeared during the treatment with hypertonic saline. Hypertonic saline treatment was given on days 1–9 after the onset of severe hyponatremia and fludrocortisone starting from day 12. In between (on days 10 and 11), patient received no treatment since the acquisition of the fludrocortisone from hospital pharmacy took 2 days. Quick increase of the serum sodium level into the steady normal range was achieved only with fludrocortisone 0.1 mg b.i.d.

In the emergency department, treatment of hyponatremia was initiated according to the current guidelines by administration of hypertonic saline via continuous infusion that resulted in improvement of patient's general condition and neurological status [4, 10]. This treatment was continued for several days at the department of oncology. However, with hypertonic saline infusion, only minor correction of hyponatremia was achieved with an increase of the serum sodium level up to only 119 mmol/L. After this slight increase in blood sodium level, an aggravation of hyponatremia evolved (115 mmol/L) with a new episode of disorientation and mental confusedness that was compared to the first episode (at admission to the emergency department) much slighter and shorter. Subsequently, the saline infusion therapy was discontinued and treatment with fludrocortisone (Florinef 0.1 mg b.i.d.) started. The treatment with fludrocortisone resulted in a quick increase in the serum sodium levels from 115 to 121 mmol/L within 3 days and subsequent persistent increase up to 131 mmol/L within the following 11 days. Changes in the serum sodium levels during the treatment of hyponatremia are illustrated in Figure 1.

The patient continued treatment with fludrocortisone (Florinef 0.1 mg b.i.d.) with no more episodes of severe hyponatremia and neurological symptoms. Eventually, due to pneumonia, second-line chemotherapy was postponed and terminated later on because of treatment brake that was long enough to allow the tumour to progress. The patient died of progressive SCLC approximately 2 months later.

## 3. Discussion

There are several conditions that result in decreased serum sodium levels in SCLC patients. Out of these, the paraneoplastic syndrome of inadequate antidiuretic hormone (ADH) secretion (SIADH, Schwartz-Bartter syndrome) is the most frequently described [4, 11]. Other possible causes of hyponatremia include ADH release during the initial lysis of the tumour cells after an effective chemotherapy, chronic heart failure, nephritic syndrome, volume depletion, and adrenal or hypophyseal deficiency [7, 12]. Also, in euvolemic patients, an increased release of ADH can be caused by several medications, such as diuretics, angiotensin-converting-enzyme (ACE) inhibitors, and selective serotonin reuptake inhibitors [7]. Our patient was euvolemic, did not have comorbidities, and was devoid of any medications that might have caused changes in his serum sodium levels. The most probable reason of severe hyponatremia in our patient was ADH release during the initial lysis of tumour cells after chemotherapy, since acute symptoms developed 2 days after the start of second-line therapy with topotecan. Similar “tumour lysis syndrome” and acute development of hyponatremia have been described in SCLC patient after the first course of chemotherapy with vindesine, ifosfamide, and cisplatin [12]. Also, in the review of 350 SCLC patients, the authors mentioned only one patient with normal sodium at the diagnosis, who subsequently developed a hyponatremia of as low as 101 mmol/L after one cycle of chemotherapy [11]. Although this phenomenon is very uncommon, it is important to be aware of this possibility because the occurrence of hyponatremia during SCLC chemotherapy could be interpreted as an increase in paraneoplastic expression reflecting tumour progression, which could in turn erroneously lead to discontinuation of the still effective chemotherapy [12].

In euvolemic patients, severely symptomatic hyponatremia should be initially corrected with continuous infusion or bolus of hypertonic saline [4]. The treatment with hypertonic saline should be stopped once the symptoms related to hyponatremia resolve, a safe blood sodium concentration is achieved, or the maximum sodium correction limits are approached [4]. In our patient, the initial acute neurological symptoms (disorientation, mental confusion) resolved; however, hyponatremia was corrected only to a maximum level of 119 mmol/L. This slight increase was followed by the secondary decrease in the sodium level (down to 115 mmol/L) and the new episode of disorientation. In such circumstances, pharmacological intervention was needed.

For the pharmacotherapy of hyponatremia, demeclocycline, a tetracycline analogue, can be administered at a dose of 100 mg to 300 mg for 3-4 times a day. Demeclocycline negates the effects of ADH at the level of renal tubules; however, the effect of this drug may be delayed for 1-2 weeks [4, 13]. Also, vasopressin receptor antagonists may be used. In particular, vasopressin receptor 2 (V2) is an attractive target of ADH, because ADH activates the V2 receptor to stimulate water reabsorption with the consequent dilution of serum sodium concentrations. Out of the vasopressin receptor antagonists, conivaptan, tolvaptan, and mozavaptan have been introduced into clinical practice, whereas newer drugs (e.g., lixivaptan, satavaptan) are in clinical testing [4]. Recently, the first report was published on a prospective case series showing an efficient management of hyponatremia with tolvaptan in patients with SCLC and severe SIADH (plasma sodium <125 mmol/L) [14]. Although tolvaptan is registered by the State Agency of Medicines of Estonia, it is not currently marketed. Therefore, we were not able to use this drug in our patient.

Out of all available medicines, we chose fludrocortisone for the treatment of severe hyponatremia in our SCLC patient. Fludrocortisone acetate is a mineralocorticoid that enhances renal tubular sodium absorption [13]. It has been used to treat several conditions that cause hyponatremia, such as adrenal insufficiency, cerebral salt wasting syndrome, and subarachnoid haemorrhage [15–17]. Moreover, successful management of hyponatremia with fludrocortisone has been reported in a patient with adrenal insufficiency caused by breast cancer bilateral adrenal metastases [18]. It has been found that oral or intravenous use of fludrocortisone at 0.2 mg b.i.d. reduces the frequency of a negative sodium balance and natriuresis and corrects thereby hyponatremia [13]. Oral fludrocortisone, at a slightly lower dose (0.1 mg b.i.d.), was also effective in the treatment of severe hyponatremia in our patient.

To the knowledge of the authors, there are no other reports that would have described the use of fludrocortisone to treat severe hyponatremia in SCLC patients. Since hyponatremia is an independent factor of poor prognosis in patients with SCLC, correction of this electrolyte abnormality should be always attempted, especially in acute situations as described in this report [6–8]. No prospective data from randomized clinical trials exist to show that correction of hyponatremia leads to prolonged survival of SCLC patients. Nevertheless, large scale retrospective study in 395 SCLC patients has shown that sodium supplementation restored normal sodium values in 91% of patients with severe hyponatremia (sodium <129 mmol/L) resulting in median survival time of 11 months [7]. In contrast, patients with falling sodium levels despite adequate treatment were prone to rapid disease progression with median survival below 3 months.

In summary, the present case demonstrated that fludrocortisone may be effectively used to correct serum sodium levels in appropriate SCLC patients. However, problems with the use of fludrocortisone, such as hypokalemia, fluid overload, and hypertension, must be carefully monitored [13].
